# Retinal artery occlusion does not act as an independent marker of upcoming dementia: results from a Danish 20-year cohort study

**DOI:** 10.1186/s40942-023-00488-3

**Published:** 2023-08-29

**Authors:** Anna Rebien Clausen, Lonny Stokholm, Morten Blaabjerg, Katrine Hartmund Frederiksen, Frederik Nørregaard Pedersen, Jakob Grauslund

**Affiliations:** 1 Department of Ophthalmology, Odense University Hospital, Kløvervaenget 5, Odense, 5000 Denmark; 2 Department of Clinical Research, University of Southern Denmark, Odense, Denmark; 3 OPEN—Open Patient data Explorative Network, Odense University Hospital, University of Southern Denmark, Odense, Denmark; 4 Department of Neurology, Odense University Hospital, Odense, Denmark

**Keywords:** Alzheimer’s disease, Dementia, Ocular biomarkers, Register-based study, Retinal artery occlusion, Vascular dementia

## Abstract

**Purpose:**

Retinal artery occlusion (RAO) is a vision threatening disease associated with cerebral vascular dysfunction, which may reflect initial signs of cerebral pathology. Early detection of patients in risk of dementia could allow for preventative treatment. Hence, this study aimed to investigate RAO as an independent biomarker of incident dementia.

**Methods:**

This study was a nationwide, 20-year longitudinal cohort study in Denmark with inclusion from 1998 to 2020 and follow up until the end of 2022. We identified 2 205 159 individuals aged 65 or older through the Danish national health registers and monitored RAO (exposure) and dementia (outcome) status. We calculated incidence rate and performed a Cox regression analysis with hazard ratio (HR) and 95% confidence interval (CI) for RAO as a marker of dementia in a crude, a semi-adjusted (age and sex), and a fully adjusted model (furthermore adjusted for marital status and systemic comorbidity.)

**Results:**

We identified 8 863 individuals with RAO. Incidence rates were higher among exposed compared to unexposed individuals (12.28 and 8.18 per 1000 person-years at risk, respectively). Individuals with RAO were more likely to be male and older at inclusion, to have hypertension, dyslipidaemia, cardiovascular disease, chronic kidney disease, and diabetes (p < 0.001). RAO was not associated with all-cause dementia in the crude analysis (HR 1.07 CI [1.00-1.17]) or in the fully adjusted analysis (HR 0.98 CI [0.91–1.06].

**Conclusion:**

Although individuals with RAO had a higher incidence of dementia compared to unexposed individuals, these associations were lost when confounders were taken into account.

**Supplementary Information:**

The online version contains supplementary material available at 10.1186/s40942-023-00488-3.

## Background

Retinal artery occlusion (RAO) is a sight threatening disease that affects 1–2 per 100 000 per year [1] and is associated with cardiovascular risk factors [1]. Dementia is a broad term encompassing a number of diseases with the common characteristic of cognitive decline. The disease burden is large with more than 55 million people affected worldwide [2], with the most frequent form being Alzheimer’s disease (AD) followed by vascular dementia (VD) [3]. Although treatment options for dementia have traditionally been limited, recent data have provided hope for treatment of early stages of AD [4]. The diagnosis is often delayed due to slow disease progression, and we need biomarkers for earlier diagnosis and a thorough understanding of disease development. The cerebral and retinal microvasculature share embryological origin and physiological properties. Vascular pathology is present in dementia, which could indicate that RAO is associated with dementia [5, 6]. This association has previously been evaluated in two smaller studies, but these show conflicting results [7, 8]. Also, previous studies included retinal vein occlusion and RAO as exposure, thus no former studies have investigated RAO alone as an independent marker of dementia. Hence, the aim of this register-based national cohort study was to investigate RAO as an independent marker of dementia (all-cause dementia, AD, and VD) amongst all Danish citizens older than 65 years.

## Methods

### Data sources

We extracted data from three Danish National Health registers. All Danish citizens are registered in the Civil Registration System by a unique personal identification number [9]. The register provides information about date of birth, age, sex, marital, and vital status, and the identification number enables linkage of information between registers. The Danish National Patient Register contains information on all somatic patient hospitalizations along with examinations and treatments since 1977. Psychiatric hospitalizations, emergency department, and outpatient contacts have also been included in the register since 1995 [10]. Diagnoses are registered according to the eight revision of International classification of Diseases (ICD) until 1994 and tenth revision from 1994 forward. The Danish National Prescription Registry holds data on prescription drugs sold in Danish pharmacies registered according to the Anatomical Therapeutic Chemical Classification (ATC) [11], which we used to define comorbid conditions.

### Study design

This longitudinal, register-based cohort study was a part of the Danish Excellence Centre in Ophthalmic Epidemiology (DECODE-EYE) study [12]. All Danish citizens aged 65 and older between sampling start (1 January 1998) and end of inclusion (31 December 2020) were included in the study. Entry date was defined by sampling start (older than 65 the 1 January 1998) or the date of the 65th birthday if this occurred within the inclusion period. End of study period was 31 December 2022.

### Exposure

Individuals with a RAO diagnosis (ICD 10 H340-2*) in the study period were registered as exposed. To account for changes over time, we considered the exposure as a time-varying variable. This means that individuals switched from the unexposed to the exposed group on the date their RAO was first registered.

Outcomes.

The primary outcome of this study was all-cause dementia, which included AD, VD, mixed, and unspecified dementia (ICD 10 F00*, F01*, G30*, F03*). Secondary outcomes were AD (ICD 10 G30.0, G30.1, G30.9, F00.0, F00.1, F00.9 ) and VD (ICD 10 F01.0, F01.2, F01.3, F01.8, F01.9 ) as isolated endpoints.

Exclusion criteria.

Individuals with any unspecified retinal vascular occlusion (ICD 10 H349) or dementia (ICD 8 290.0, 290.1, 293.0, 293.1, 337.02-3 and ICD 10 G30*, F00*, F01*, F02*, or F03) registered in The Danish National Patient Register before entry date were excluded from the study.

### Covariates

We included covariates in our analysis based on a priori knowledge, including age in 5-year categories, sex, marital status (never married, married/cohabiting, or divorced/widowed), and various medical conditions including hypertension, dyslipidaemia, cardiovascular disease, chronic obstructive pulmonary disease (COPD), diabetes, and chronic kidney disease. The definition of medical conditions including diagnostic codes (ICD 8 and ICD 10) and ATC-codes were based on previous research [13–15]. The definitions are available in supplementary Table 1.

We considered marital status and sex as fixed at the entry date, while hypertension, dyslipidaemia, diabetes, chronic kidney disease, and cardiovascular disease were regarded as time-varying variables that could change status during the follow-up period. We adjusted for COPD if it was ever registered, as this served as a proxy for smoking exposure, which we considered too imprecise to be included as a time-varying variable.

### Statistical analysis

Baseline characteristics are presented as numbers and percentages and tested for differences using chi-square tests.

We calculated incidence rate (IR) and reported the primary outcome as hazard ratio (HR) with 95% confidence interval (CI) in a crude, a semi-adjusted (age and sex), and a fully adjusted (all covariates) Cox proportional hazards regression model of individuals exposed to RAO compared to unexposed individuals. Individuals were followed from entry date until the date of (i) the first dementia diagnosis, (ii) emigration, (iii) death, or (iv) end of follow up (31 December 2022), whichever came first.

We censored individuals with a registration of an unspecified retinal vascular occlusion (ICD 10 H349) the day before the diagnosis was registered. Similarly, we censored individuals with dementia in other diseases classified elsewhere (ICD 10 F02*), as their outcome status are unreliable with the other dementia form blurring a potential subsequent AD or VD diagnosis.

### Supplementary analyses

We included specialty requirements on RAO and dementia in a sensitivity analysis to accommodate the risk of misclassification by incorrect diagnostic code registration. We censored individuals with a registration of RAO in other than an ophthalmologic or a neurologic department the day before diagnosis registration. Likewise, dementia diagnoses registered at either a neurologic, geriatric, psychiatric, or internal medicine department were considered valid.

Danish clinical guidelines include examination for a carotid artery stenosis following a RAO and surgical intervention in case of significant stenosis [16, 17]. This intervention is considered a preventative procedure for development of dementia. We tested for interaction with sex and registration of carotid endarterectomy operation (KPAF20-21, KPAQ20-21, and KPAP20-21) after occurrence of RAO.

We performed the analyses using Stata version 17.0 (StataCorp LLC, College Station, TX, USA).

## Results

**Fig. 1:**
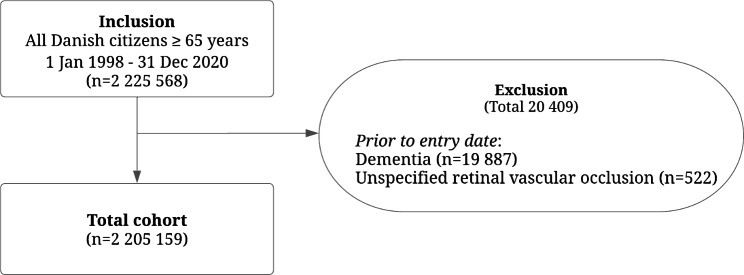
Flowchart of the study individuals

Figure 1 illustrates the selection of the study population. The final cohort was comprised of 2 205 159 individuals, of which 8 863 individuals were registered with a RAO during the study period. Of the entire study population, approximately 75% were between 65 and 70 years old at inclusion and median follow up time was 9.72 years (interquartile range 5.13–14.92) (data not shown).

The individuals exposed to a RAO were more likely to be male, older at entry, less frequently married, and more prone to hypertension, dyslipidaemia, diabetes, chronic kidney disease, and cardiovascular comorbidity at entry date (p < 0.001). The exposed individuals had more frequently underwent a carotid endarterectomy (p < 0.001), and only a tendency to different occurrence of COPD (p = 0.05) (Table 1).

**Table 1:** Baseline characteristics

	All n = 2 205 159	RAO exposed n = 8 863	Never exposed n = 2 196 296	P-value
Sex, n (%)				
Female	1 180,743 (53.5)	4 245 (47.9)	1 176 498 (53.6)	< 0.001
Male	1 024 416 (46.5)	4 618 (52.1)	1 019 798 (46.4)	< 0.001
Age at entry, n (%)				< 0.001
65–70 years	1 642 770 (74.5)	6 341 (71.5)	1 636 429 (74.5)	
70–75 years	199 574 (9.1)	1 068 (12.1)	198 506 (9.0)	
75–80 years	163 768 (7.4)	788 (8.9)	162 980 (7.4)	
80 + years	199 047 (9.0)	666 (7.5)	198 381 (9.0)	
Civil status, n (%)				< 0.001
Never married	793 230 (36.0)	3 317 (37.4)	789 913 (36.0)	
Married/cohabiting	1 135 534 (51.5)	4 337 (48.9)	1 131 197 (51.5)	
Widowed	276 395 (12.5)	1 209 (13.6)	275 186 (12.5)	
Comorbidity conditions, n (%)				
Hypertension	844 824 (38.3)	4 674 (52.7)	840 150 (38.3)	< 0.001
Dyslipidaemia	358 872 (16.3)	2 084 (23.5)	356 788 (16.2)	< 0.001
Diabetes	159 876 (7.3)	945 (10.7)	158 931 (7.2)	< 0.001
COPD	63 273 (2.9)	285 (3.2)	62 988 (2.9)	0.050
Chronic kidney disease	11 786 (0.5)	101 (1.1)	11 685 (0.5)	< 0.001
Cardiovascular comorbidity	262 296 (11.9)	2 046 (23.1)	260 250 (11.8)	< 0.001
Procedure, n (%)				
Carotid endarterectomy	2 026 (0.1)	84 (0.9)	1 942 (0.1)	< 0.001

Baseline characteristics of the study population, the exposed and unexposed groups at entry date. Data are presented as number and percentages

COPD, Chronic obstructive pulmonary disease; RAO, retinal artery occlusion

P-value for ${\chi }^{2}$ test

IR and HR for individuals exposed to and unexposed to RAO are presented in Table 2. IR are generally higher in the exposed group compared to the unexposed group.

**Table 2:** Hazard Ratio and 95% Confidence Interval for dementia according to retinal artery occlusion status

	Exposed		Unexposed		Crude model	Model adjusted for age and sex	Fully adjusted model**
	No of events / PYR	IR*	No of events / PYR	IR*	HR (95% CI)	HR (95% CI)	HR (95%CI)
All-cause dementia	678 / 56 421	12.28	188 16 / 22 996 806	8.18	1.07 (1.00-1.16)	1.09 (1.01–1.18)	0.98 (0.91–1.06)
Alzheimer’s disease	248 / 58 058	4.27	81 069 / 23 390 830	3.42	0.93 (0.82–1.06)	0.92 (0.81–1.04)	0.93 (0.82–1.05)
Vascular dementia	140 / 58 557	2.39	26 915 / 23 653 110	1.12	1.61 (1.36–1.90)	1.61 (1.36–1.90)	1.12 (0.95–1.33)

Number of events, incident rates and hazard ratios for incident dementia for patients with and without exposure

PYR, person-years at risk; IR, incidence rate; HR, hazard ratio; CI, confidence interval

*Per 1000 person-years

**Model adjusted for age, sex, marital status, and systemic comorbidity (hypertension, diabetes, chronic kidney disease, chronic obstructive pulmonary disease, and dyslipidaemia

Individuals with a RAO had no higher risk of developing all-cause dementia (fully adjusted HR 0.98, 95% CI 0.91–1.06), nor AD. Individuals with RAO had a higher risk of VD compared to unexposed individuals (HR 1.61, 95% CI 1.36–1.90), but this association was not present in the fully adjusted model accounting for confounding variables (HR 1.12, 95% CI 0.95–1.33). For all-cause dementia and VD, it is noticeable that the HR did not vary much between the crude and semi-adjusted model, but changed significantly when systemic comorbidity was taken into account.

The sensitivity analysis with requirements for departments to register the diagnoses showed no different result (supplementary Table 1) and we found no interaction with carotid endarterectomy or sex.

## Discussion

In this nationwide cohort study, we found an increased incidence and unadjusted hazard of VD amongst individuals exposed with RAO compared to unexposed. While this was not affected by age and sex, associations were no longer statistically significant, once systemic comorbidity was taken into account. Hence, we conclude that the included comorbidities can be considered shared risk factors of retinal and cerebral vascular disease. The vascular systems in the retina and cerebrum are interconnected and both neural organs are sensitive for interruptions in oxygen supply. This warrants that diseases affecting one vascular system may have implications for the other, as demonstrated in this paper.

We found no association between RAO and all-cause dementia or AD.

The association between RAO and dementia is not previously studied with RAO as an exclusive exposure, although two studies have examined RAO in combination with retinal vein occlusion as a marker of dementia [7, 8]. The first study, a single-institution study of 37,208 individuals investigated the association between retinal vascular occlusions and dementia in a cross sectional analysis [8]. Our results were in alignment with this study, but further comparisons are hindered by difference in study design. The second study was an American cohort study including 4 743 individuals with 1 102 cases of dementia [7]. Individuals in the study with apolipoprotein ε4 genotype had three-fold risk of VD when exposed to a retinal vascular occlusion, and they reported no association among non-apolipoprotein ε4 carriers. No association between retinal vascular occlusion and AD was reported [7]. As a limitation of our study, we could not test this interaction, as we did not have any data on genome predisposition. Likewise, we had no information about life style factors as exercise, diet, smoking, and alcohol. For the latter, we were, however, able to include COPD as a proxy for smoking.

The strengths of this study included the study design, as this was a register-based cohort study with a long follow up time of an entire nation with no loss to follow up. This is the largest study to date to investigate RAO as a marker of dementia and the first study to examine RAO as an isolated exposure. Furthermore, no other study have examined the association between RAO and dementia in Scandinavian citizens.

Although RAO was not demonstrated to be an independent marker of dementia, the increased risk of vascular dementia in the crude analysis demonstrates a clinically important association between RAO and dementia. Thus, patients with a RAO have higher systemic comorbidity than patients without RAO and consequently higher risk of dementia.

## Conclusion

In this nationwide, register-based cohort study, individuals with a RAO had no independently increased risk of all-cause dementia, AD or VD. However, we demonstrated an increased risk of VD ascribed to the shared risk factors with RAO.

### Electronic supplementary material

Below is the link to the electronic supplementary material.

**Supplementary table 1**: Codebook of diagnostic codes (ICD 8 and 10) and ATC codes applied in this study.

**Supplementary table 2**: Results from supplementary analysis. Cox regression analysis with requirements on departments to register diagnoses.

## Data Availability

Data cannot be shared due to the General Data Protection Regulation.

## Abbreviations

AD: Alzheimer’s disease
ATC: Anatomical Therapeutic Chemical Classification
CI: Confidence interval
COPD: Chronic obstructive pulmonary disease
HR: Hazard ratio
IR: Incidence rate
RAO: Retinal artery occlusion
PYR: Person-years at risk
VD: Vascular dementia
