# Supplementary material for: Retinal artery occlusion does not act as an independent marker of upcoming dementia: results from a Danish 20-year cohort study
Source: Int J Retina Vitreous. 2023 Aug 29;9:50. doi: 10.1186/s40942-023-00488-3 (PMC10466746; doi:10.1186/s40942-023-00488-3)
Supplement: Supplementary file 1 — Supplementary table 1: Codebook of diagnostic codes (ICD 8 and 10) and ATC codes applied in this study. [file 40942_2023_488_MOESM1_ESM.pdf]

Supplementary table 1: Codebook of diagnostic codes (ICD 8 and 10) and ATC codes applied in this study.

ICD 10 codes used for exposure

**Illness**	**ICD 10**	**Criteria**
Retinal artery occlusion	H340*, H341*, H342*	ICD 10 code registered once. Date of first registration counts for date of exposure.

ICD 10 codes used for outcomes

**Illness**	**ICD 10**	**Criteria**
All-cause dementia	F00*, F01*, G30*, F03*	Any ICD 10 code registered once. Date of first registration counts for date of outcome.
Alzheimer's disease	G30.0, G30.1, G30.9, F00.0, F00.1, F00.9	
Vascular dementia	F01.0, F01.2, F01.3, F01.8, F01.9	

ICD 8, ICD 10, and ATC codes used for covariates

**Illness**	**ICD 8**	**ICD 10**	**ATC**	**Criteria**
Diabetes mellitus	249, 250	DE10	A10A	DE10 given before the 30th birthday AND at least 1 prescription of A10A
		DE11		2x DE11
		DE12, DE13, DE14		Any registration of DE12, DE13, or DE14
			A10A, A10B	2 prescriptions of A10A or/and A10B
Dyslipidaemia[a]		E78	C10	Any registration of E78<br>Two prescriptions of ATC C10 within 12 months<br>WITHOUT a previous registration of ICD I21*-I22*, I240*-I241*, I248 or I249*
COPD[a]		J44*		J44 only as A-diagnosis and if patient is >30 years at diagnosis
		J96*		J96 as A-diagnosis AND ONLY in combination with J44 as B-diagnosis
		J13*-18*		J13-18 as A-diagnosis AND ONLY in combination with J44 as B-diagnosis
Hypertension[b]		I10*, I11, I119*, I12*,I13, I131, I139, I15	α adrenergic blockers (C02A*, C02B*, C02C*)<br>non-loop diuretics (C02DA*, C02L*, C03A*, C03B*, C03D*, C03E*,	ICD 10 and/or ATC, including all prescriptions with a combination treatment with at least two of the drug classes of antihypertensive drugs with ATC codes.

		C03X*, C07C*, C07D*, C08G*, C09BA*, C09DA*, C09XA52)<br>vasodilators (C02DB*, C02DD*, C02DG*)<br>ß blockers (C07*, MC09BX02, MC09BX04, MC09BX05)<br>calcium channel blockers (C07FB*, C08*, C09BB*, C09DB*)<br>renin-angiotensin system inhibitors (C09*)	
Chronic kidney disease	N18, N19		Either N18 or N19
Cerebrovascular disease[c]			Criteria from Charlson Comorbidity Index Including congestive heart failure, peripheral vascular disease, and cerebrovascular disease WITHOUT DH340
Carotid endarte-rectomy	KPAQ20,<br>KPAQ21,<br>KPAF20,<br>KPAF21,<br>KPAP20,<br>KPAP 21		Any ICD 10 KPA* diagnosis

ICD, International classification of diseases; ATC, Anatomical Therapeutic Chemical Classification System; COPD, chronic obstructive pulmonary disease.

*including subcodes

[a]As defined by Frederiksen KH, L Stokholm, PH Frederiksen, CM Jorgensen, S Moller, R Kawasaki, T Peto & J Grauslund (2022): Cardiovascular morbidity and all-cause mortality in patients with retinal vein occlusion: a Danish nationwide cohort study. Br J Ophthalmol.

[b]As defined by Hvidberg MF, SP Johnsen, C Glümer, KD Petersen, AV Olesen & L Ehlers (2016): Catalog of 199 register-based definitions of chronic conditions. Scand J Public Health 44: 462-479.

[c]Thygesen SK, CF Christiansen, S Christensen, TL Lash & HT Sørensen (2011): The predictive value of ICD-10 diagnostic coding used to assess Charlson comorbidity index conditions in the population-based Danish National Registry of Patients. BMC Med Res Methodol 11: 83.
