# Supplementary material for: Retinal artery occlusion does not act as an independent marker of upcoming dementia: results from a Danish 20-year cohort study
Source: Int J Retina Vitreous. 2023 Aug 29;9:50. doi: 10.1186/s40942-023-00488-3 (PMC10466746; doi:10.1186/s40942-023-00488-3)
Supplement: Supplementary file 2 — Supplementary table 2: Results from supplementary analysis. Cox regression analysis with requirements on departments to register diagnoses. [file 40942_2023_488_MOESM2_ESM.pdf]

Supplementary table 2: Cox regression analysis with requirements on departments to register diagnoses.

	Exposed		Unexposed		Crude model	Model adjusted for age and sex	Fully adjusted model**
	No of events / PYR	IR*	No of events / PYR	IR*	HR (95 % CI)	HR (95 % CI)	HR (95 %CI)
All-cause dementia	544 / 51 137	10.64	169 683 / 22 996 755	7.38	1.06 (0.97-1.15)	1.07 (0.99-1.17)	0.97 (0.90-1.06)
Alzheimer's disease	205 / 52 628	3.90	81 004 / 23 390 811	3.14	0.93 (0.801-1.06)	0.91 (0.79-1.04)	0.93 (0.81-1.06)
Vascular dementia	117 / 53 035	2.21	26 920 / 23 653 105	1.06	1.58 (1.32-1.90)	1.59 (1.32-1.90)	1.12 (0.93-1.34)

Number of events, incident rates and hazard ratios for incident dementia for patients with and without exposure

PYR, person-years at risk; IR, incidence rate; HR, hazard ratio; CI, confidence interval.

*Per 1000 person-years

**Model adjusted for age, sex, marital status, and systemic comorbidity (hypertension, diabetes, chronic kidney disease, chronic obstructive pulmonary disease, and dyslipidaemia
